# Active Site Loop Conformation Regulates Promiscuous Activity in a Lactonase from *Geobacillus kaustophilus* HTA426

**DOI:** 10.1371/journal.pone.0115130

**Published:** 2015-02-23

**Authors:** Yu Zhang, Jiao An, Guang-Yu Yang, Aixi Bai, Baisong Zheng, Zhiyong Lou, Geng Wu, Wei Ye, Hai-Feng Chen, Yan Feng, Giuseppe Manco

**Affiliations:** 1 State Key Laboratory of Microbial Metabolism, School of Life Sciences and Biotechnology, Shanghai Jiao Tong University, Shanghai, People’s Republic of China; 2 Key Laboratory for Molecular Enzymology and Engineering of Ministry of Education, Jilin University, Changchun, People’s Republic of China; 3 Environmental Science Research and Design Institute of Zhejiang Province, Hangzhou, People’s Republic of China; 4 Laboratory of Structural Biology, School of Medicine, Tsinghua University, Beijing, People’s Republic of China; 5 Institute of Protein Biochemistry, National Research Council, Naples, Italy; Weizmann Institute of Science, ISRAEL

## Abstract

Enzyme promiscuity is a prerequisite for fast divergent evolution of biocatalysts. A phosphotriesterase-like lactonase (PLL) from *Geobacillus kaustophilus* HTA426 (*Gka*P) exhibits main lactonase and promiscuous phosphotriesterase activities. To understand its catalytic and evolutionary mechanisms, we investigated a “hot spot” in the active site by saturation mutagenesis as well as X-ray crystallographic analyses. We found that position 99 in the active site was involved in substrate discrimination. One mutant, Y99L, exhibited 11-fold improvement over wild-type in reactivity (*k_cat_*/*K_m_*) toward the phosphotriesterase substrate *ethyl*-paraoxon, but showed 15-fold decrease toward the lactonase substrate δ-decanolactone, resulting in a 157-fold inversion of the substrate specificity. Structural analysis of Y99L revealed that the mutation causes a ∼6.6 Å outward shift of adjacent loop 7, which may cause increased flexibility of the active site and facilitate accommodation and/or catalysis of organophosphate substrate. This study provides for the PLL family an example of how the evolutionary route from promiscuity to specificity can derive from very few mutations, which promotes alteration in the conformational adjustment of the active site loops, in turn draws the capacity of substrate binding and activity.

## Introduction

Enzyme promiscuity can function as a starting point in divergent evolution for generating a specific enzyme in the presence of selective stress. A better understanding of catalytic promiscuity can improve our knowledge of protein evolution and ancestry as well as providing new tools for protein engineering and biotechnological applications [1–3]. One of the most important models for studying enzyme promiscuity is the enzyme that degrades synthetic organophosphate (OP) compounds, including most agricultural pesticides and chemical warfare agents, which first appeared on this planet during the 20th century [4]. Currently, enzymatic detoxification of OP compounds has become an important subject worldwide, because continuous and excessive use of OP compounds has led to the contamination of many terrestrial and aquatic ecosystems [5]. Indeed, most OP degrading enzymes are known promiscuous enzymes [6–9]. One typical example is phosphotriesterase (PTE, EC 3.1.8.1) from the soil bacteria *Brevundimonas* (formerly *Pseudomonas*) *diminuta* (*bd*PTE), which has high catalytic efficiency for hydrolyzing a variety of neurotoxic OP compounds [10, 11]. The same protein and a closely related protein with 90% sequence identify were identified in *Flavobacterium sp*. (OPH) [12] and *Agrobacterium radiobacter* (OpdA) [13], respectively. In addition to a wide substrate spectrum of OP substrates, *bd*PTE was recently reported to possess promiscuous lactonase and esterase activities [6]. Furthermore, a number of PTE remote homologs have been characterized from several microorganisms and shown to proficiently hydrolyze various lactones with weak PTE activity [14–19]. These new enzymes that emerged were designated as PTE-like lactonases (PLL) [14] and were ascribed to a new family in the amidohydrolase superfamily [14, 18]. It has been hypothesized that PTEs evolved from an unknown member of the PLL family in response to changing environmental conditions [14]. Promiscuous activities in PLL members have been successfully enhanced by molecular evolution in the laboratory. The obtained mutants, which possess several mutational sites, showed a significant increase in PTE activities [20–23]. However, to date, the detailed evolutionary relationships between PLL enzymes and PTE enzymes has not been fully understood.

The three-dimensional structures of several PLL enzymes have been solved previously [16–20]. Structural comparison of the PLLs with *bd*PTE [24] showed that the overall structures superpose very well. All of these enzymes belong to the amidohydrolase superfamily and possess an essentially identical binuclear metal center embedded within the (β*/*α)_8_-TIM barrel fold [25]. Remarkably, PLLs differ from *bd*PTE in the length and the topology of surface loops 7 and 8. Both loops 7 and 8 in the amidohydrolase superfamily are most often found in contact with the bound substrates and playing important role in determination of substrate specificity [26]. A chimeric PLL enzyme from *Deinococcus radiodurans* (*Dr*OPH) containing these additional loops from *bd*PTE did not exhibit enhanced PTE activity as expected, most likely due to the difficulty to replicate in a different environment the features provided by a specific loop [17, 20]. Amino acid Trp131 in the active site of PTEs is conserved, while at the same position in PLLs a Tyr has been found. The aromatic side chain of Trp131 in *bd*PTE plays an important role in the interaction with the leaving aromatic ring of the OP substrate through π-stacking interactions, which facilitates substrate binding to the active site [27]. However, the tyrosine residue in PLLs appears to stabilize the lactone ring during catalysis, and a mutant Y97W in the PLL enzyme from *Sulfolobus solfataricus* (*Sso*Pox) showed a 3-fold increase in PTE activity [18]. Indeed, the crystal structure of *Geobacillus kaustophilus* HTA426 (*Gka*P) shows that the binding site for the putative leaving group is lined with a series of very hydrophobic residues, including Tyr99, Tyr100, Trp271, and Pro275. These findings suggested that Tyr99 of *Gka*P may be involved in substrate discrimination. However, so far no study has explored this conserved tyrosine residue in PLL family members.

In this study, a PLL enzyme from the thermophilic bacterium *Geobacillus kaustophilus* HTA426 (*Gka*P) was characterized. To explore its promiscuous catalytic mechanism, a saturation mutagenesis library at position 99 was constructed and screened for enhanced PTE activity. The structural factors controlling both the catalytic efficiency and substrate specificity were further identified by crystal structure analysis and molecular docking. These results showed that position 99 in the active site of PLLs could be evolved to develop greater PTE’s substrate specificity. Importantly, the structural analysis indicated that the dramatic movement of the active loop 7 of *Gka*P provides a catalytically competent active site architecture to account for the catalytic enhancements observed in the evolved mutant. This work shows that active site loop conformation regulates promiscuous activity and alters the capacity for both substrate binding and efficient catalysis.

## Results and Discussion

### Sequence and structure similarity

**Fig 1:**
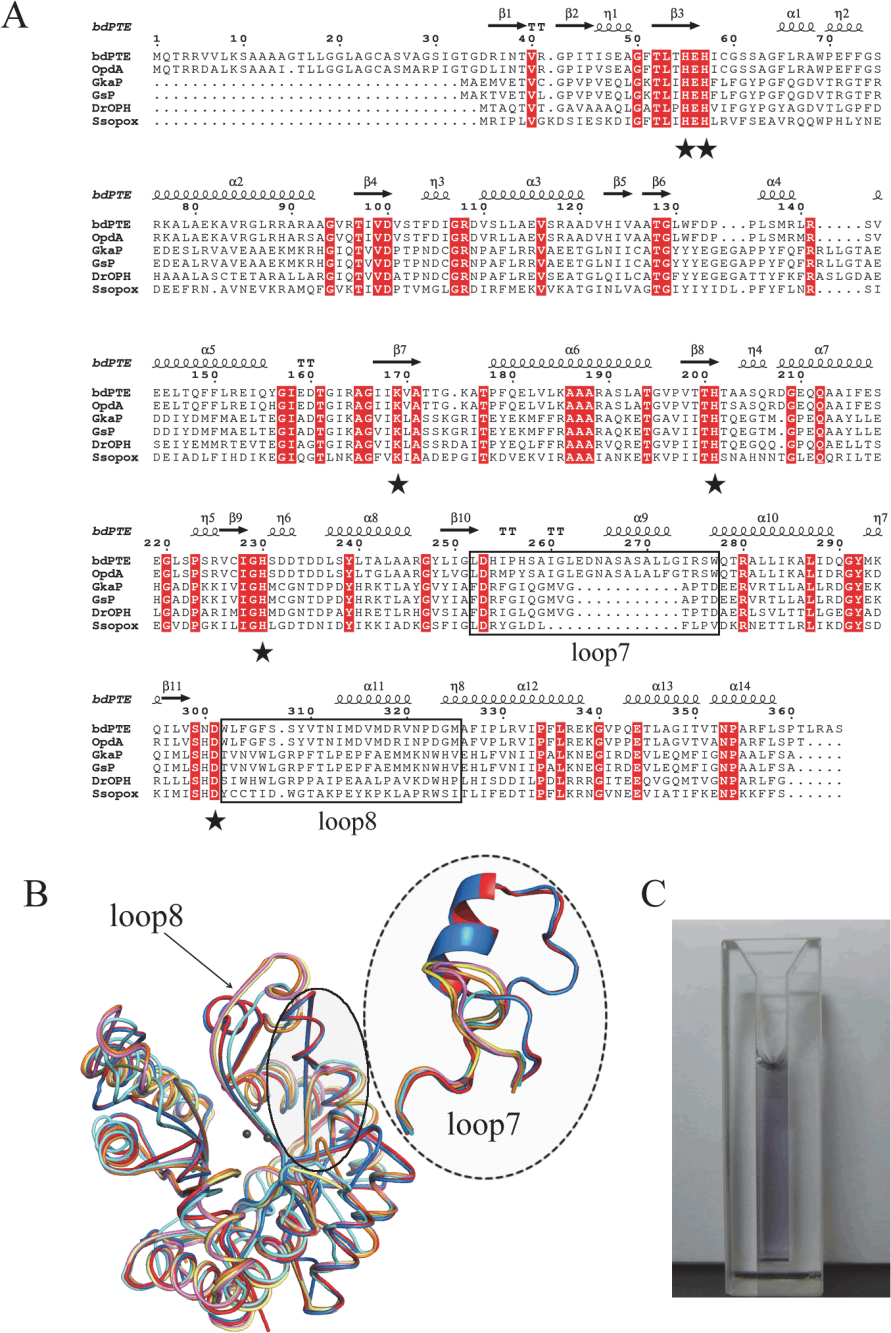
Sequence, structure and color of *Gka*P. (A) Structure-based sequence alignment between *Gka*P (gi|56420041) and other phosphotriesterase (PTE) homologous proteins. A structure-based sequence alignment was performed using ClustalW and ESPript (http://espript.ibcp.fr). The relationships of these sequences to those found in PTE from *Brevundimonas diminuta* (*bd*PTE; gi|2098312), PTE from *Agrobacterium radiobacter* (OpdA; gi|15212234), PLL from *Geobacillus stearothermophilus* (GsP; gi|15899258), PLL from *Deinococcus radiodurans* (*Dr*OPH; gi|15805954), and PLL from *Sulfolobus solfataricus* (*Sso*Pox; gi|15899258) are shown for comparison. Conserved residues that constitute the binuclear metal center are labeled in asterisks. The loop 7 and loop 8 regions are indicated in the black square. (B) Superposition of *Gka*P (yellow) with other related PTE homologous protein structures. *bd*PTE (red) (PDB ID 1DPM), OpdA (blue) (PDB ID 2D2G), GsP (pink) (PDB ID 3F4D), *Dr*OPH (orange) (PDB ID 3FDK), and *Sso*Pox (cyan) (PDB ID 2VC7). (C) Visible purple color for purified *Gka*P. The protein concentration is approximately 8 mg/mL.

The structural-based sequence alignment of the *Gka*P gene with the reported PLLs and PTEs is shown in Fig. 1A. *Gka*P is most closely related to the PLL from thermophilic *Geobacillus stearothermophilus* (GsP) with an identity of 98%. It also showed sequence identity to the PLL from the extremely radioresistant bacterium *Deinococcus radiodurans* (*Dr*OPH) and PLL from hyperthermophilic archaeon *Sulfolobus solfataricus* (*Sso*Pox) with an identity of 58% and 33%, respectively. In comparison to PTEs, *Gka*P was distantly related to mesophilic *bd*PTE and OpdA, with sequence identities of 24% and 25%, respectively. The sequence alignment indicated that *Gka*P contains entirely conserved residues (His23, His25, Lys145, His178, His206 and Asp266) within the binuclear metal center. The structure of the *Gka*P (PDB ID 3TN3) was also compared to the PLL and PTE members (Fig. 1B). All structures possess a (β/α)_8_-barrel fold and a binuclear metal center, which is typical of the amidohydrolase superfamily. Both loop 7 and loop 8, which are hypothesized to bind substrates of the amidohydrolase superfamily [26], overhang the binuclear metal center. Superposition of the structure of *Gka*P with those of PLL and PTE members produced the RMSD of all C_α_ atoms ranging from 0.272 to 1.084 Å, indicating that these proteins are spatially homologous. Similar to other PLL enzymes, the length of loop 7 in *Gka*P is 12 residues shorter than *bd*PTE and OpdA, and loop8 in *Gka*P has almost the same length as in *bd*PTE and OpdA, but its topology structure is more detached from the protein core than that of *bd*PTE and OpdA. Previous studies have presumed that the elongation of loop 7 in *bd*PTE forms a short α-helix, which may provide an active “cap” that narrows the active site mouth and may thereby increase PTE preference toward the substrate paraoxon [14].

The *Gka*P gene was cloned into pET-28a (+) with an N-terminal His_6_-tag and subsequently overexpressed in *E. coli* BL21 (DE3). The recombinant protein was expressed at a high yield (∼50 mg/L) in the 2YT medium containing 1 mM CoCl_2_. The purified Co^2+^-containing *GkaP* appeared purple colored (Fig. 1C), as also observed in another PLL enzyme, *Dr*OPH, that has 58% homology to *Gka*P. It is noteworthy that this purple color was different than the brown color that was observed with Co^2+^-containing GsP, though both proteins only differed by four residues in the N-terminal tail. Since the coloring was attributed to a charge transfer between an active site tyrosine residue and *β*-metal complex [17, 28], these proteins may have substantial variations in the microenvironment of the active site due to the long distance interactions, despite a homology as high as 98%.

### Catalytic promiscuity of *Gka*P

We next tested *Gka*P activities against lactonase, phosphotriesterase, and esterase substrates, respectively. The hydrolytic activities of the recombinant *Gka*P against an array of lactones were detected and the values of the kinetic parameters are summarized in Table 1. We found that the enzyme efficiently hydrolyzes six-membered ring lactones containing a hydrophobic side chain. The best substrate was found to be *δ*-decanolactone, with *k*_*cat*_, *K*_m_, and *k*_*cat*_ */K*_m_ values of 68.08 s^-1^, 0.069 mM, and 9.9 × 10^5^ s^-1^M^-1^, respectively. The kinetic curve showed that the enzyme reached a saturated state quickly at low substrate concentration**∼**0.5 mM (S1A Fig.). Similar to other PLL enzymes, *Gka*P also exhibited weak activity toward *ethyl*-paraoxon, which is a typical PTE substrate. This was exemplified by the fact that the saturation kinetic curve was not obtainable up to a 5 mM concentration of *ethyl*-paraoxon (S1B Fig.). The catalytic efficiency (*k*_*cat*_ */K*_m_) was estimated to be 100 s^-1^M^-1^ under the pseudo-first-order condition ([s]<<*K*_m_), which is over 100-fold higher than that measured for the PLL *Dr*OPH (*k*_*cat*_ */K*_m_ 0.83 s^-1^M^-1^) [17]. In addition, PLL members have been shown to catalyze the ester hydrolysis [15]. We also detected a significant esterase activity for *Gka*P with *p*-nitrophenyl caprylate, which had a *k*_cat_/K_m_ of 1.3× 10^3^ s^-1^M^-1^(Table 1; S1C Fig.). The activity analysis demonstrated that *Gka*P was catalytically promiscuous and had proficient lactonase activity similar to currently reported PLLs (*k*_*cat*_ */K*_m_), which was in the range of 10^5^–10^6^ s^-1^M^-1^ [14, 17]. Similar to other PLLs, *Gka*P exhibited weak PTE activity, which was approximately 10^5^-fold lower than *bd*PTE.

**Table 1:** Kinetic parameters for *Gka*P at 75°C.

Substrate		*k*_cat_	*K*_m_	*k*_cat_/*K*_m_
		s^-1^	mM	s^-1^M^-1^
**Lactone**	*γ*-Hexyl-γ-butyrolactone	3.7±0.2	0.051±0.009	7.3×10^4^
	*δ*-Valerolactone	53.7±2.5	5.6±0.7	9.6×10^3^
	*δ*-Nonalactone	56.6±1.2	0.11±0.01	5.1×10^5^
	*δ*-Decanolactone	68.1±1.6	0.069±0.006	9.9×10^5^
	*ε*-Caprolactone	34.0±3.1	16.2±2.7	2.1×10^3^
**Carboxyl ester**	*p*-Nitrophenyl caprylate	0.9±0.04	0.68±0.06	1.3×10^3^
**Phosphotriester**	*Ethyl*-paraoxon	ND^a^	ND^a^	1.1×10^2^
	*Methyl*-paraoxon	ND^a^	ND^a^	38.0

^a^ Not determined, saturation kinetics could not be attained up to a 5mM concentration. Only the catalytic efficiency (*k*_cat_/*K*_m_) can be estimate under pseudo first-order conditions ([S]*<< K*_m_).

### Enhancement of PTE activity in *Gka*P

To enhance the promiscuous PTE activity of *Gka*P, the residue contributing to substrate binding was selected and mutated. The conserved Trp131 of *bd*PTE, which is a residue located at the entrance of the binuclear metal center, has been reported to participate in substrate binding through hydrophobic interactions between its indole ring and the aromatic ring of the paraoxon [27, 29]. In PLL members, a conserved tyrosine is present at the position that corresponds to Trp131 in *bd*PTE/OpdA. This residue is important for lactonase activity, because the hydroxyl group forms hydrogen bonds with the carbonyl group of the lactone substrate and stabilizes the lactone ring in a favorable state during catalysis [18]. Therefore, we mutated the Tyr99 residue of *Gka*P by saturated mutagenesis to incorporate all 20 possible amino acids in order to assess if a specific residue could increase the PTE activity. We constructed a small mutation library with approximately 200 clones. All clones with obvious activity changes were sequenced, which provided specific mutants of interest for further purification and enzymatic characterization.

The hydrophobic Leu, Trp, and Phe residues were chosen for further characterization because they induced a substantial increase in PTE activity (up to 11-fold). In addition, we choose Ala, which has the smallest side chain and was used as a minimum reference to study the function of the other side chains. The saturation kinetic curves of the mutants for *ethyl*-paraoxon were not obtainable, with the exception of the Y99W mutant (S2 Fig.). The catalytic efficiency (*k*_*cat*_ */K*_m_) were estimated under the pseudo-first-order condition ([s]<<K_m_) and are detailed in Table 2. The mutant Y99A had a 2-fold decrease in *k*_cat_/*K*_m_, which suggested that the volume and polarity of the side chain at position 99 is important for the catalysis. The Y99W mutant exhibited the smallest *K*_m_ value compared to WT and other mutants, which is beneficial for substrate binding. However, its overall 5-fold increase in *k*_cat_/*K*_m_ suggests that this substitution may affect *ethyl*-paraoxon hydrolysis. The Y99F mutant also exhibited a 2-fold improvement in *k*_cat_/*K*_m_. The kinetic analysis revealed that Leu at position 99 provided the largest increase in PTE activity, and mutant Y99L displayed an 11-fold enhancement in *k*_cat_/*K*_m_ toward *ethyl*-paraoxon. These results indicated that the hydrophobic side chain substitutions, such as leucine, phenylalanine, or tryptophan, were favored at position 99 in order to sustain the activity against a phosphotriester substrate.

**Table 2:** Kinetic parameters of *Gka*P wild type and mutants with phosphotriesterase or lactonase activity^a^.

Enzyme	*Ethyl*-paraoxon				*δ*-Decanolactone			
	*k*_cat_ (s^-1^)	*K*_m_ (mM)	*k*_cat_/*K*_m_ (s^-1^M^-1^)	Fold efficiency	*k*_cat_ (s^-1^)	*K*_m_ (mM)	*k*_cat_/*K*_m_ (s^-1^M^-1^)	Fold efficiency
Wild type	ND^b^	ND^b^	1.1×10^2^	1	68.1±1.6	0.069±0.006	9.9×10^5^	1
Y99L	ND^b^	ND^b^	1.2×10^3^	10.9	12.7±0.4	0.20±0.02	6.4×10^4^	0.06
Y99W	0.7±0.03	1.3±0.1	5.4×10^2^	4.9	26.5±0.9	0.20±0.03	1.3×10^5^	0.13
Y99I	ND^b^	ND^b^	5.7×10^2^	5.2	17.2±0.6	0.16±0.04	1.1×10^5^	0.11
Y99V	ND^b^	ND^b^	72	0.7	16.6±0.8	0.26±0.05	6.4×10^4^	0.06
Y99F	ND^b^	ND^b^	2.3×10^2^	2.1	14.7±0.7	0.15±0.02	9.8×10^4^	0.99
Y99A	ND^b^	ND^b^	56	0.5	19.1±1	0.23±0.04	8.3×10^4^	0.08

^a^ All assays at 75°C.

^b^ Not determined, saturation kinetics could not be attained.

All of the Y99X mutants had reduced lactonase activity as measured by δ-decanolactone hydrolysis, which showed significantly elevated *K*_m_ values (2–3 fold) toward *δ*-decanolactone and a 3–5-fold decrease in *k*_cat_ values relative to wild-type (Table 2). The finding that all of these mutants had an increase in *K*_*m*_ suggests that the hydroxyl group of Tyr99 plays a crucial role in binding of the substrate to the wild-type enzyme. The Y99X mutants showed increased PTE activity and decreased lactonase activity. A more substantial effect occurred with mutant Y99L, which caused a specificity switch of 157-fold. These results unambiguously confirm the importance of position 99 in substrate discrimination by *Gka*P.

**Fig 2:**
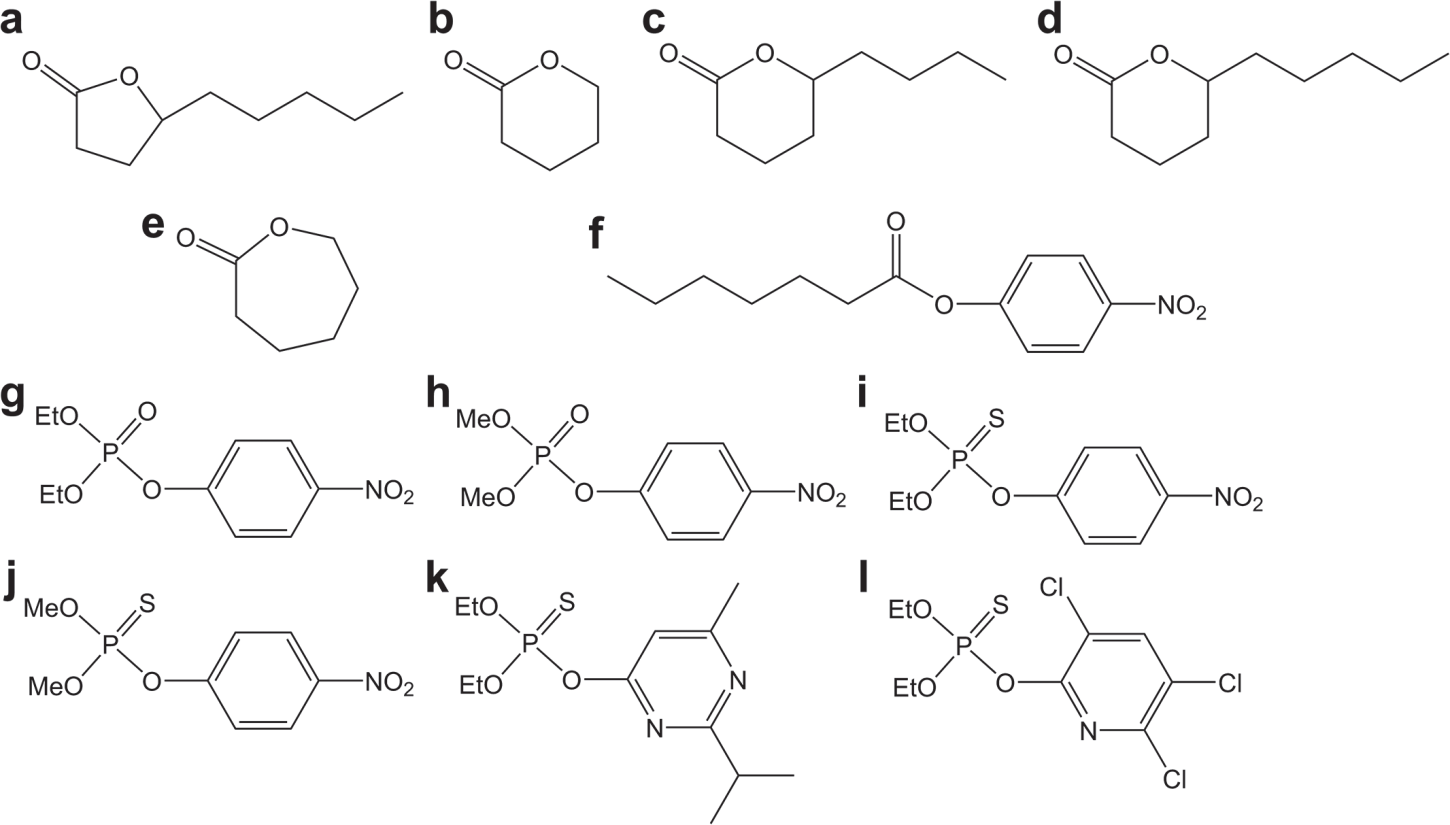
Structures of the substrates used in this study. (a) *γ*-hexyl-*γ*-butyrolactone; (b) *δ*-valerolactone; (c) *δ*-nonalactone; (d) *δ*-decanolactone; (e) *ε*-caprolactone; (f) *p*-nitrophenyl caprylate; (g) *ethyl*-paraoxon; (h) *methyl*-paraoxon; (i) *ethyl*-parathion; (j) *methyl*-parathion; (k) diazinon; (l) chlorpyrifos.

Because *bd*PTE mainly hydrolyzes OP pesticides, a variety of pesticides (Fig. 2, g-l) were used to test the activities of the active mutants described above (Table 3). Both wild-type and mutants favored pesticide substrates with diethyl side chains. The specific activity of the Y99W mutant against all of the pesticides was 2–4-fold higher than the wild-type enzyme. Strikingly, the mutant Y99L dramatically enhanced catalytic activity toward all pesticides from 4 to 90-fold. It is noteworthy that the activity improvement for thiophosphoryl pesticides is much higher than that for the phosphoryl pesticides. While the activity of Y99L increased 4- to 6-fold for the *methyl*-paraoxon and *ethyl*-paraoxon, the enhancement was 70- to 90-fold for their thiophosphoryl counterpart (*methyl*- or *ethyl*-parathion). Therefore, Tyr99 in *Gka*P can evolve enzymatic function and markedly enhance the weak PTE activity.

**Table 3:** Specific activities of the wild-type *Gk*aP and mutants with several organophosphate pesticides.

OP pesticide	Specific activity (mU/mg)^a^		
	WT	Y99W	Y99L
*Ethyl*- paraoxon	242.0±4.5	499.5±4.2	1035.1±67.8
*Methyl*-paraoxon	61.9±5.9	164.7±18.2	392.2±55.4
*Ethyl*- parathion	15.5±0.5	37.7±2.2	1072.5±34.0
*Methyl*-parathion	5.6±0.4	21.2±1.7	519.7±40.5
Diazinon	36.4±0.3	38.6±4.5	1882.2±56.6
Chlorpyrifos	1.5±0.2	3.6±0.7	7.3±1.4

^a^ One unit (U) of enzymatic activity was defined as the amount of enzyme that catalyzes the hydrolysis of 1μmol substrate per minute at 75°C.

### X-ray structures and analysis of the wild-type *Gka*P and mutant Y99L

**Fig 3:**
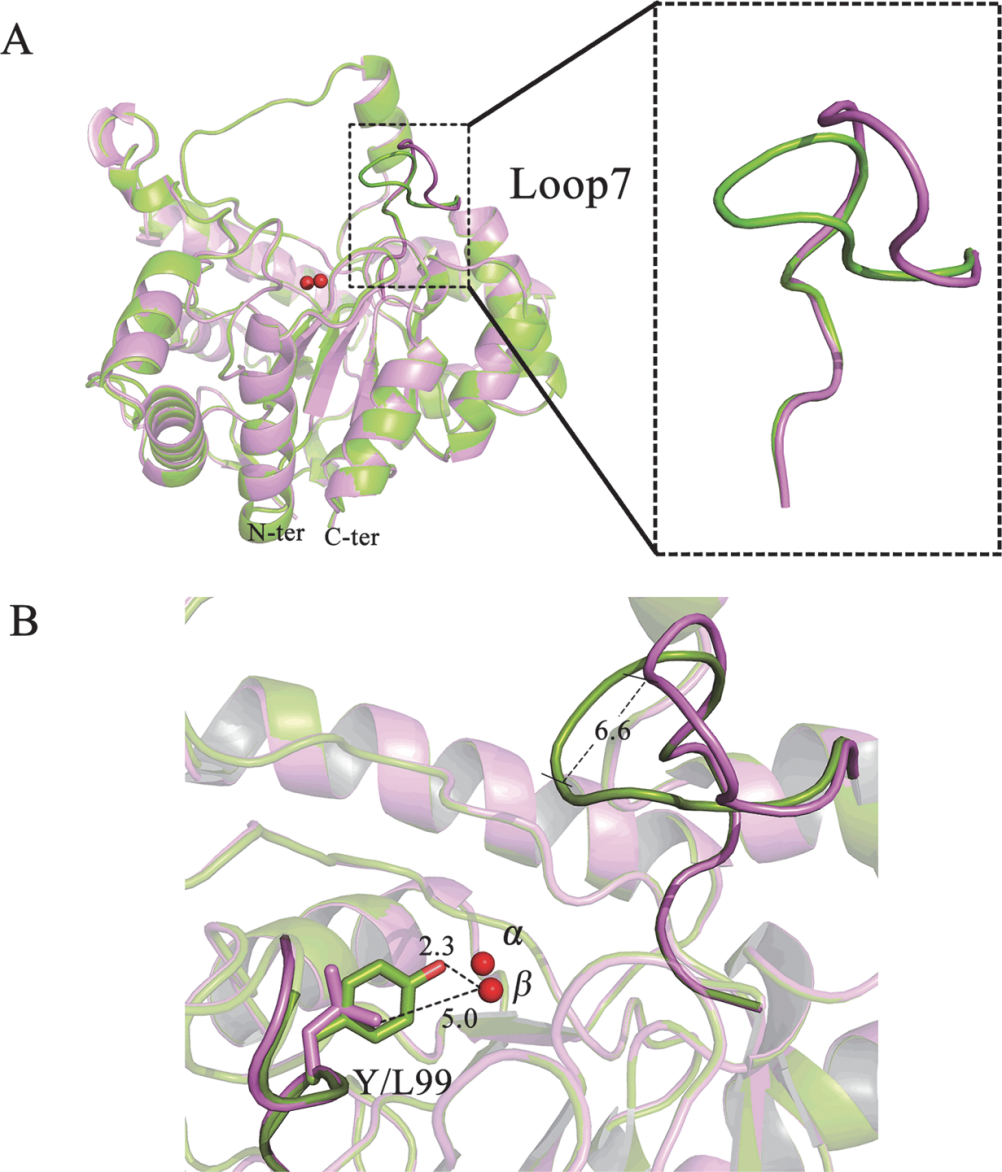
Crystal structure analysis of *Gka*P wild-type and mutant Y99L. (A) Overlay of the structures of wild-type *Gka*P (green), and Y99L (purple). (B) Overlay of loop 7 regions of wild-type *Gka*P (green) and Y99L (purple). The metal ions are shown in red spheres. Distances are shown in Å.

To gain a better understanding of the structural basis for the changes in both catalytic spectra and activity caused by the mutations, X-ray crystal structures of the wild-type protein and mutant Y99L were solved at a resolution between 1.50 and 1.75 Å. The details of the data collection and structure refinement are summarized in Table 4. The overall structure of *Gka*P contained an (β/α)_8_ barrel fold, which is in agreement with the structures of other PLL enzymes. Compared to the structure of wild-type *Gka*P, there were no major structural changes induced by these mutations. The root mean square deviation (RMSD) for all backbone atoms between the structures were all under 0.3 Å. The main structural feature that was noticeably different between the mutants was in the conformational variability of loop 7 (Fig. 3A). The overlaid structures of Y99L and wild-type *Gka*P showed a conspicuous outward shift of the active site in the region of loop 7 by a distance of approximately 6.6 Å (Fig. 3B). This loop contains several residues (Arg230, Ile233, Met236, and Val237) that are found within a large binding pocket and may function in substrate specificity. A recent study has proposed that remote mutations in OpdA result in significant changes in the conformational distribution of loop 7. The dominant open conformation of loop 7 may provide the proper steric space for binding OP substrates. The closed state is optimally pre-organized for paraoxon hydrolysis, but seems to block access to the active site [30]. We identified an open conformation of loop 7 in Y99L mutant. From the structural analysis, replacement of Tyr99 with Leu created an extra 2.7 Å space in the distance between the C_δ_ of Leu99 and the *β*-metal (Fig. 3B). An explanation could be the vanishing of the interaction between Arg230 and Tyr99. In the wild type enzyme, a single water molecule at position 713 was found to form two hydrogen bonds with the hydroxyl group of Tyr99 (2.5 Å) and the guanidine group of Arg230 (3.2 Å), respectively (S3 Fig.). This hydrogen-bonded bridge may stabilize the conformation of loop 7 and kept it at close state. However the Y99L mutation disrupts the hydrogen-bonded network, which in turn pushes the flexible Arg230 against residues of loop 7 favoring the open conformation. The loop movement causes expansion of the substrate binding pocket volume to 953 Å^3^ as calculated by CASTp online, which is approximately double the volume of the wild-type *Gka*P space (430 Å^3^). In addition, the hydrophobic Leu side chain may increase the affinity for phosphotriester substrates in the binding site.

**Table 4:** Data collection and refinement statistics for *Gka*P wild type and mutant.

Structure	w.t. *Gka*P	Y99L
**Data collection statistics**		
Cell parameter: a, b, c (Å)	51.31, 88.15, 89.99	51.36, 80.30, 92.20
Space group	*P*2_1_	*P*2_1_
Wavelength used (Å)	0.97916	0.97916
Resolution (Å)^a^	50.0–1.6(1.66–1.6)	50.0–1.75(1.81–1.75)
No. of molecule/asymmetric unit	2	2
No. of unique reflections	99136	74050
No. of all reflections	706232	552363
Completeness (%)^a^	95.8 (93.2)	99.8 (100)
Redundancy	7.1(6.8)	7.5(7.4)
I/σ (I)	34.6 (3.7)	13.1 (3.6)
*R*_*merge*_ ^*b*^ (%)	5 (43.1)	13.2 (47.5)
**Refinement statistics**		
*R*_*work*_ ^*c*^ (%)	18.12	22.19
*R*_*free*_ (%)	19.82	24.93
RMSD bond lengths (Å)	0.0067	0.0057
RMSD bond angle (°)	1.0636	0.8796
Overall B-factor (Å^2^)	24.42	27.376
Final model (Number of protein atoms)	5093	5088
Final model (H_2_O molecules)	738	505
**Ramachandran plot**		
Res. in most favored regions	625	627
Res. in additionally allowed regions	10	11
Res. in generously allowed regions	0	0

^a^ Values in parentheses correspond to the highest resolution shell.

^b^ *R*_*merge*_ = Σ_*hkl*_Σ_*i*_|*I(hkl)*_*i*_-<*I(hkl)*>|/Σ_*hkl*_Σ_*i*_I*(hkl)*_*i*_, where <*I(hkl)*> is the mean intensity of the observations *I(hkl)*_*i*_ of reflection hkl.

^c^ *R*_work_ = Σ||*F*_*obs*_||*F*_*calc*_||/Σ|*F*_*obs*_|, where *F*_*obs*_ and *F*_*calc*_ are the observed and calculated structure-factor amplitudes, respectively. *R*_free_ was calculated as *R*_work_ using a randomly selected subset of ∼10% of unique reflections not used for structure refinement.

**Fig 4:**
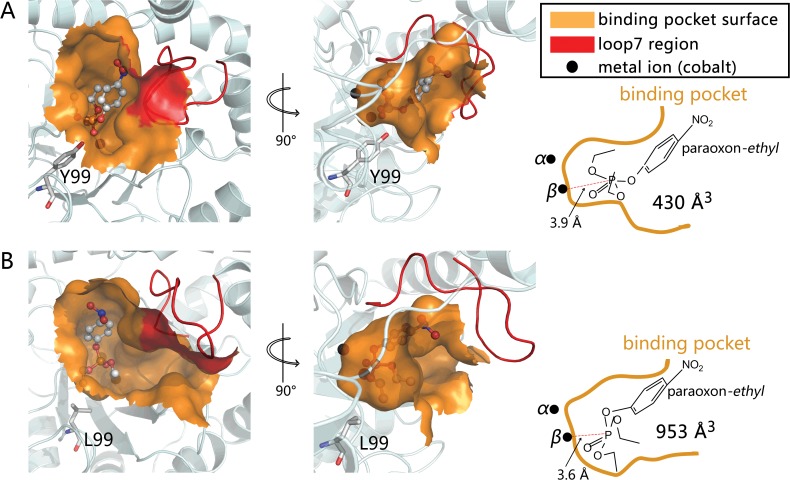
Binding model of *ethyl*-paraoxon into the active site of wild-type *Gka*P (A), and Y99L (B). Dockings were performed using AutoDock 4.0 on a rigid protein structure. Binding pockets are shown at the surface. Loop 7 is shown in red. The cobalt ions are labeled as *α* and *β*. Substrate *ethyl*-paraoxon is shown as ball-and-stick. Y99 and L99 are shown as stick models (gray carbons).

To qualitatively estimate the significant enhancement in the activity of the *Gka*P mutant Y99L, a structure analysis and *in silico* docking experiments were conducted. The results indicated that the distance between the phosphorus atom of *ethyl*-paraoxon and the *β*-cobalt ion is 3.85 Å in the wild-type (Fig. 4A), but 3.6 Å in the Y99L mutant, respectively (Fig. 4B). In contrast to the wild-type binding model, the phosphorus center of the substrate in the Y99L mutant models are closer to the *β*-cobalt ion, which would result in strong polarization of the P-O bond and benefit catalysis.

To gain a better understanding for the preference of Y99L mutant for the thiophosphoryl pesticides substrates (Table 3), we performed substrate docking and 10 ns’ MD simulations on Y99L complex with *ethyl*-paraoxon (P = O) and diazinon (P = S) bound, respectively. The binding free energies are -10.83±3.19 kcal/mol for Y99L-paraoxon and -22.36±4.37 kcal/mol for Y99L-diazinon, which confirmed that the mutant Y99L did favors P = S over the phosphoryl pesticides substrates. However, the structural basis of this change is not clear and need further research to reveal.

### Concluding remarks

In a previous paper [21] some of us reported the effect of mutations Phe and Leu at position Trp263 and Trp at Tyr97 as able to increase the PTE activity and specificity in *Sso*Pox, a finding suggested to be related to the widening of the leaving group subsite. This has been confirmed by recent structural analyses showing that W263X induces very subtle changes in the loop 8 positioning whereas have no effect on the very short loop 7 [31]. Here we demonstrated that mutagenesis of the Tyr99 in the active site of *Gka*P can markedly enhance its promiscuous PTE activity. This residue presumably contributes to the process of the natural divergent evolution in the PTE family. The structural analyses revealed that the enhancement of enzyme activity could be caused by alterations in the dynamics and flexibility of substrate-binding loop 7, which creates a wide and open state of the active site. The transition between the close (wild type) and open (mutant) conformational states governed by loop 7 movement is directly associated with the regulation of the substrate specificity. Our study provides an example that a limited mutation in a promiscuous enzyme may lead to structural changes and benefit for an alternative binding substrate and efficient catalysis. It also suggests that the conformation of active site loop plays an essential role for regulating promiscuous activity of the enzyme, reinforcing the need to consider the interplay between active site architecture and catalysis in future protein engineering.

## Materials and Methods

### Reagents, bacterial strains, and plasmids

All substrates were purchased from Sigma-Aldrich (Shanghai, China), and the chemical structures are shown in Fig. 2. Restriction enzymes, T4 DNA ligase, LA TaqDNA polymerase, kanamycin, and isopropyl 1-thio-β-D-galactopyranoside (IPTG) were obtained from Fermentas (Vilnius, Lithuania) and TaKaRa (Kyoto, Japan). The molecular mass marker for SDS-PAGE was obtained from Fermentas. Ni-NTA agarose was purchase from Qiagen (Chatsworth, CA). All other chemicals were reagent grade. *Geobacillus kaustophilus* HTA426 was purchased from the Japan Collection of Microorganisms (JCM No.12893). The expression vector pET-28a (+) as well as *E. coli* strainsXL1-blue and BL21-CodonPlus (DE3)-RIL, which were used for cloning and protein expression, respectively, were purchased from Invitrogen (Carlsbad, CA).

### Cloning of the GK1506 gene and DNA manipulation

Standard molecular cloning and transformation techniques were employed as described by Sambrook *et al* [32]. The gene encoding a PLL (GenBank ID: 3183579) was amplified by PCR from *Geobacillus kaustophilus* HTA426 genomic DNA using the primers listed in S1 Table. PCR conditions were 95°C for 5 min, followed by 30 cycles at 95°C for 40 s, 56°C for 40 s, 72°C for 90 s, and a final extension at 72°C for 8 min. The amplified gene was cloned into the pET-28a (+) vector with an N-terminal 6×His tag. The recombinant plasmid was then transformed into *E. coli* XL1-blue electrocompetent cells and plated onto 2YT agar plate containing 50 μg/mL kanamycin overnight. The insert sequence was confirmed by DNA sequencing.

### Expression and purification of *Gka*P

The recombinant plasmid was transformed into *E. coli* BL21-CodonPlus (DE3)-RIL electrocompetent cells. Cells were grown at 37°C in 2YT medium supplemented with 50 μg/mL kanamycin and 1.0 mM CoCl_2_ until the OD_600_ reached 0.6–0.8. Protein expression was then induced by the addition of 1.0 mM IPTG and the culture was grown for 12 h at 26°C. Cells were harvested by centrifugation and resuspended in 50 mM Tris-HCl (pH8.0) containing 0.2 mM Co^2+^ and 5 mM imidazole. After ultrasonic cell disintegration, the cytosolic fraction was heated at 60°C for 30 min and centrifuged at 12000 rpm for 20 min to remove heat-induced protein aggregates. The supernatant was added to a Ni^2+^-chelating affinity column and eluted with 100 mM imidazole in 20mM Tris-HCl (pH 7.9) and 10 mM NaCl for an approximate total of 10 mL. The purity of the fusion enzymes (wild type and mutant) was assessed on 15% SDS-PAGE and enzymes were diafiltrated twice with 50 mM Tris-HCl (pH 8.0).

### Kinetics measurements of *Gka*P

The lactonase, phosphotriesterase, and esterase hydrolyses of *Gka*P were monitored by absorbance changes in a UV-2550 spectrophotometer (Shimadzu, Kyoto, Japan) at a constant temperature of 75°C with 1 mL reaction volumes (path length = 1cm). Analysis of reaction samples for each substrate was performed at a constant enzyme concentration. The *δ*-decanolactone substrate was dissolved in dimethyl sulfoxide (DMSO), whereas the *p*-nitrophenyl caprylate and OP substrates in acetonitrile as stock solutions. For enzymatic kinetics assay, aliquots of the stock were added to the reaction buffer for defined concentrations. The hydrolysis of lactone was monitored using a pH-sensitive colorimetric assay [33]. Briefly, the reactions were performed in 2.5 mM Bicine (pH 8.3) containing 0.2 M NaCl, 0.2 mM cresol purple, and 0.02–20 mM lactone substrate. Upon mixture of the substrate with the enzyme, the decrease in absorbance was monitored at 577 nm (ε_577_ = 47300 M^-1^cm^-1^, 1%DMSO). The enzyme was diafiltrated with 10 mM bicine (pH 8.3), with a PD-10 column (GE Healthcare, Shanghai, China) before use. Kinetic measurements with *p*-nitrophenyl caprylate (*p*NPC8), and *ethyl*-paraoxon were performed in 50 mM phosphate buffer (pH 8.0). The reaction rates were monitored by the release of *p*-nitrophenol (ε_405_ = 16000M^-1^cm^-1^, 2% acetonitrile). The initial rates were corrected for the background rate of spontaneous hydrolysis in the absence of enzymes, which were subtracted from the enzymatic rates. The kinetic parameters (*k*_cat_, *K*_m_) were obtained by fitting the data to the Michaelis-Menten equation [V = S×E×*k*_cat_/(S+*K*_m_)] or to the pseudo-first-order form of it at S<<*K*_m_ (V = S×E×*k*_cat_/*K*_m_) with GraphPad Prism software (Graphpad, San Diego, CA), where V is the initial velocity, E is the enzyme concentration, S is the substrate concentration. The final concentrations used in the assay to determine specific activities toward the tested OP pesticides were 1mM, 1mM, 0.25 mM, and 0.25 mM for paraoxon, parathion, diazinon, and chlorpyrifos, respectively. The rate of paraoxon and parathion hydrolysis was measured at a wavelength of 405nm as described above. The rate of diazinon hydrolysis was measured at a wavelength of 228nm (ε_228_ = 3300 M^-1^cm^-1^), and chlorpyrifos was measured at 276 nm (ε_276_ = 2790 M^-1^cm^-1^). Control reactions were performed in the absence of enzyme. One unit of enzymatic activity was defined as the amount of enzyme that catalyzed the hydrolysis of 1 μmol substrate per minute. All assays were performed at least in triplicate. Enzyme concentrations were determined using the Bio-Rad protein assay kit II (Bio-Rad, Richmond, CA).

### Site-directed mutagenesis

The mutantsY99W, Y99I, and Y99V were constructed using the QuikChange site-directed mutagenesis protocol [34]. The pET-28a (+) plasmid carrying the wild-type or mutant *Gka*P gene was used as a template for PCR with LA Taq polymerase (TaKaRa). All mutagenic primers are listed in S1 Table. The PCR reactions were as follows: initial denaturation for 5 min at 94°C, followed by 16 cycles of 94°C for 30 s, 57°C for 30 s, and 72°C for 8 min. A final elongation step at 72°C was performed for 30 min. The final PCR reaction was then incubated with 2 U DpnI (Fermentas) at 37°C for 1 h to remove the methylated template, and 2 μL of the reaction was transformed into competent E. coli BL21-CodonPlus (DE3)-RIL cells. The expression and purification procedures of these mutants were the same as described for the wild-type construct.

### Site-saturation mutagenesis and screening procedure

Saturation mutagenesis at Tyr99 was performed and the primers used are listed in S1 Table. The target amino acid position was coded by NNK (forward) and MNN (reverse), where N = A, G, C, or T, K = G or T, and M = A or C. Whole plasmid PCR reactions and transformations were performed as described in the method for site-directed mutagenesis. A library of clones was screened for paraoxon hydrolysis activity compared to the activity of wild type using a 96-well plate assay. Approximately 200 randomly selected colonies were picked with sterilized toothpicks and placed into individual wells of 96-well plates, which were filled with 200 μL of 2YT containing 50 μg/mL kanamycin and 1.0 mM CoCl_2_. The plates were shaken overnight at 37°C. The next day, 5 μL of each culture was transferred into a new plate containing fresh medium and antibiotic. The original plates were stored at 4°C and the duplicated plates were shaken for an additional 3 h for cell growth. The cells were then induced with 1 mM IPTG for 6 h at 30°C, and then the cell density in each well was determined by measuring the OD at a wavelength of 600 nm using a Multiskan Ascent 96-well plate reader (Thermo Scientific, Vantaa, Finland). Cells were harvested by centrifugation at 3000 rpm for 30 min. The harvested cells were frozen and then thawed three times to release the enzyme and then resuspended in 200 μL of 50 mM phosphate buffer (pH 8.0). Crude bacterial extracts were heated at 60°C for 30 min and centrifuged in order to remove the heat-induced aggregated proteins. A 100 μL aliquot from each well was pipetted into a new 96-well plate, to which 100 μL of substrate solution containing 0.4 mM *ethyl*-paraoxon and 0.2 mM Co^2+^ in 50 mM phosphate buffer at pH 8.0 was added. Hydrolysis of paraoxon was performed for 5 min at 75°C. The absorbance was measured based on the released *p*-nitrophenol at a wavelength of 405 nm. The value of A_405_ of each well was normalized by the corresponding A_600_, and the ratio *r* = A_405_/A_600_ was used to estimate the activity of each clone.

### Crystallization of *Gka*P wild-type and mutant

Crystals of native *Gka*P and mutant were grown by the hanging-drop method at 16°C. Each drop contained 2 μL of protein at a concentration of either 20 or 30 mg/mL and 2 μL of the 200 μL well solution. The reservoir solutions for wild-type *Gk*aP, contained 50 mM Hepes-Na pH 7.3, 12–13% (w/v) PEG8000, 8% (w/v) ethylene glycol, and 2% (w/v) glycerol. For the Y99L mutant, the reservoir solution contained 0.1M MES pH 6.5 and 14% (w/v) PEG20000. Crystals were cryoprotected by the addition of 20% ethylene glycol (v/v) to the crystallization conditions. The crystals belonged to space group *P2*_*1*_. The datasets at 1.6, 1.5, 1.75, and 1.75 Å that corresponded to wild-type *Gk*aP and Y99L mutant, respectively, were collected on beamline BL17U at the Shanghai Synchrotron Radiation Facility (China). All datasets were integrated, scaled, and reduced with the HKL2000 software package. The structures were solved by Molecular Replacement with the CCP4i program PHASER using the wild-type *Gk*aP structure (PDB code 3ORW) that was previously obtained by our laboratory as the model. Model building was performed by COOT, and all of the model qualities were checked with PROCHECK.

### Substrates docking

All docking runs were performed using AutoDock 4.0. The cobalt van der Waals parameters were 2.87 Å for radii, 0.014 kcal/mol for well depth, and 12 Å^3^ for atomic salvation volume. Charges of 1.4 and 1.5 were assigned to the *α* and *β* cobalt ions, respectively, as previously described [35]. A 30×30×30 grid with 0.375Å spacing was centered in a metal center. The Lamarckian genetic Algorithm (LGA) was selected for the ligand conformational search. For the docking process, the number of generation was 20 conformers for each OP substrate. Other parameters were used as default. Among docking poses, the lowest binding energy conformation was selected. The structures were drawn with PyMOL 1.1. The residue interaction was analyzed using Ligplot 4.22. The volume of substrate binding pocket was calculated using CASTp [36] online (http://sts.bioengr.uic.edu/castp/calculation.php).

### Molecular Dynamics Simulations

Molecular Dynamics (MD) simulations and energy minimizations were performed using the AMBER12 simulation package [37] and the *ff12SB* force field [38] with the TIP3P water model [39]. The initial coordinates of the complexes were extracted from the docking results. Hydrogen atoms were added using the LEAP module of AMBER12. Antechamber [40] was used to handle the force field of *ethyl*-paraoxon and diazinon. Ten Na^+^ ions were placed around the complex to maintain the system’s neutrality. The complexes were solvated in a truncated octahedron box of water, with the solvent buffer of 10 Å. The SHAKE algorithm [41] was used to constrain bonds involving hydrogen atoms. Particle Mesh Ewald (PME) [42] was employed to calculate long-range electrostatic interactions. All the MD simulations were accelerated with the CUDA version of PMEMD [43, 44] in GPU cores of NVIDIA Tesla K20. After up to 10000 steps of minimization and 40 ps heating procedure, the systems were equilibrated at 298 K for 100 ps. Then, 10 ns’ MD simulation was employed to record time trajectory. The time step used in all calculations was 2.0 fs. Coordinates were saved every 5 ps for the purpose of subsequent analyses.

### Protein Data Bank accession codes

The coordinates have been deposited in the Research Collaboration for Structural Bioinformatics (RCSB) Protein Data Bank with the following accession codes: 3TN3 for wild-type *Gka*P, and 3TN5 for Y99L.

## Supporting Information

S1 Fig Michaelis-Menten kinetics analyses of lactonase (A), phosphotriesterase (B) and esterase (C) activities of *Gka*P. Lactonase assay used with *δ*-decanolactone; Phosphotriesterase assay used with *ethyl*-paraoxon, saturation kinetics could not be attained; Esterase assay used with *p*-nitrophenyl caprylate.
(TIF)

S2 Fig Michaelis-Menten kinetics analyses of phosphotriesterase activities of *Gka*P and mutants. *Ethyl*-paraoxon was used as substrate.
(TIF)

S3 Fig The interaction between Arg230 and Tyr99 in the wild-type *Gka*P. A water molecule (Wat713, shown in blue sphere) forms two hydrogen bonds with Tyr99 and Arg230, respectively. Tyr99 and Arg230 are shown as stick models (green carbons). The two metal ions are labeled as *α* and *β*, and shown in red spheres. Loop 7 region is highlighted in orange.
(TIF)

S1 Table Primers used in cloning and mutagenesis of *Gka*P. (DOCX)
