# Supplementary figures and images for: Active Site Loop Conformation Regulates Promiscuous Activity in a Lactonase from Geobacillus kaustophilus HTA426
Source: PLoS One. 2015 Feb 23;10(2):e0115130. doi: 10.1371/journal.pone.0115130 (PMC4338136; doi:10.1371/journal.pone.0115130)

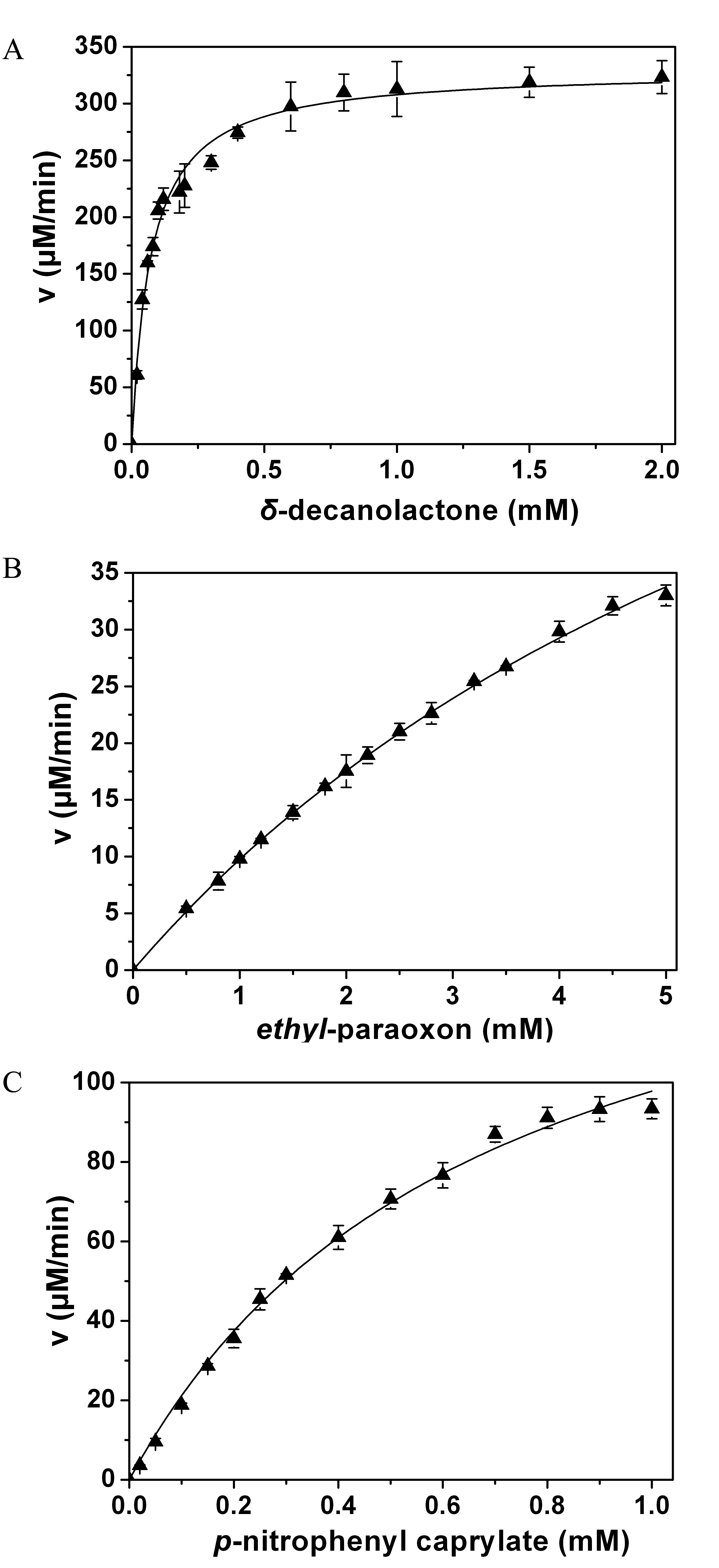

Supplement: S1 Fig — Lactonase assay used with δ-decanolactone; Phosphotriesterase assay used with ethyl-paraoxon, saturation kinetics could not be attained; Esterase assay used with p-nitrophenyl caprylate. (TIF) [file pone.0115130.s001.tif]

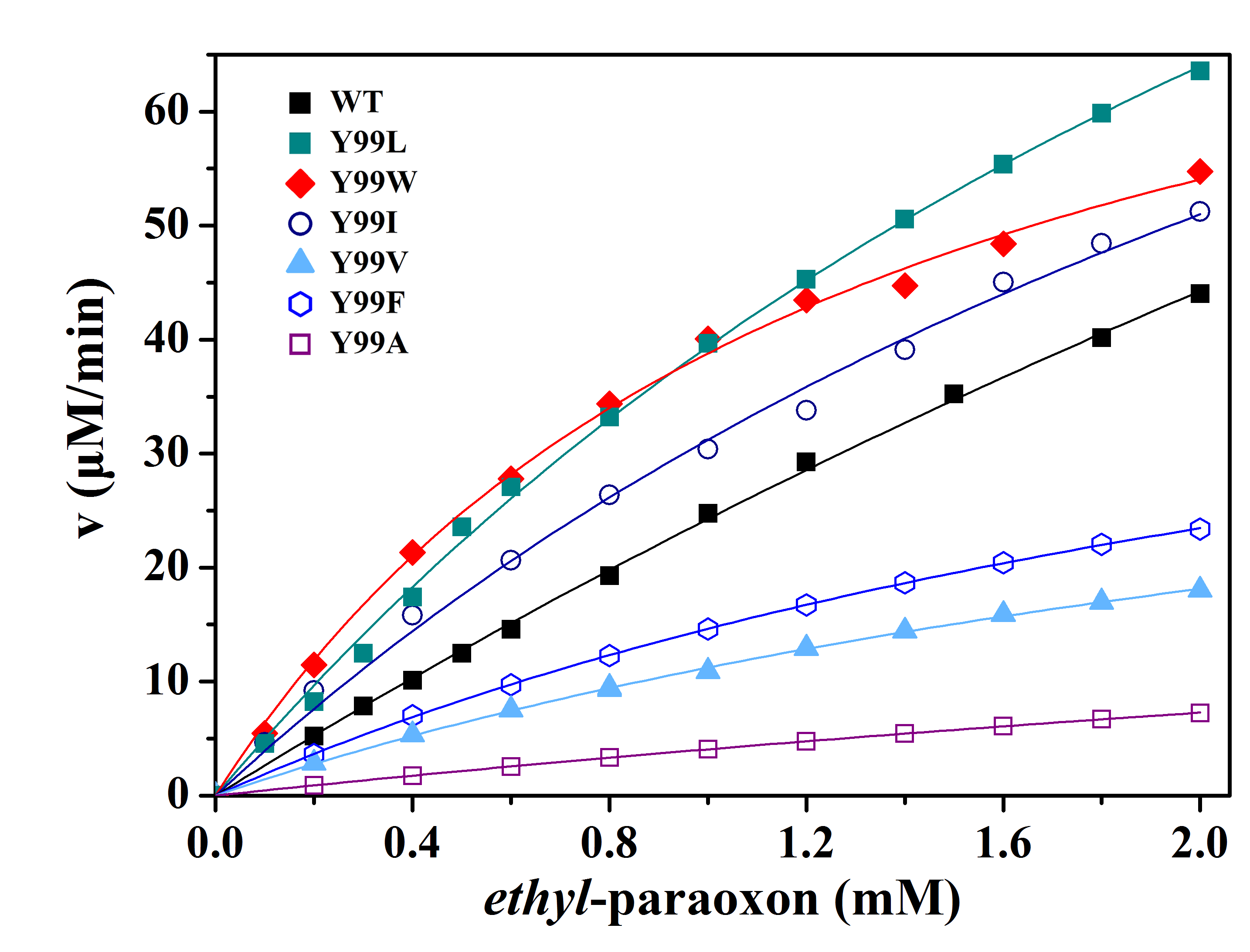

Supplement: S2 Fig — Ethyl-paraoxon was used as substrate. (TIF) [file pone.0115130.s002.tif]
