# Supplementary material for: Active Site Loop Conformation Regulates Promiscuous Activity in a Lactonase from Geobacillus kaustophilus HTA426
Source: PLoS One. 2015 Feb 23;10(2):e0115130. doi: 10.1371/journal.pone.0115130 (PMC4338136; doi:10.1371/journal.pone.0115130)
Supplement: S1 Table — (DOCX) [file pone.0115130.s004.docx]

**Table S1.** Primers used in cloning and mutagenesis of *Gka*P.

| **Primer** | **Oligonucleotide sequence (5'-3')^a^** |
| --- | --- |
| **Gene cloning** |  |
| *Gka*P (forward) | GCGC**GGATCC**ATGGCGGAGATGGTAGAAACGGTAT |
| *Gka*P (reverse) | GATC**AAGCTT**GTCAAGCCGAGAACAGCGCCGCCGGAT |
| **Site-saturation mutagenesis** |  |
| Y99X saturation (forward) | ACCGGCTATNNKTATGAAGGGGAAGG |
| Y99X saturation (reverse) | CTTCATAMNNATAGCCGGTGGCGCAA |
| **Site-directed mutagenesis** |  |
| Y99W (forward) | ACCGGCTATTGGTATGAAGGGGAAGG |
| Y99W (reverse) | GGGTCCGTGTTCCAGCACATATGGC |
| Y99I (forward) | ACCGGCTATATTTATGAAGGGGAAGG |
| Y99I (reverse) | GGGTCCGTGTTAATGCACATATGGC |
| Y99V (forward) | ACCGGCTATGTGTATGAAGGGGAAGG |
| Y99V (reverse) | GATGCCGAACACGTCAAACGCAATG |

^a^ Restriction enzyme cutting sites are indicated in bold. The nucleotide changes are underlined.
